# Bacterial Infection in Ebola Virus Disease, Marburg Virus Disease, Crimean–Congo Hemorrhagic Fever, and Lassa Fever

**DOI:** 10.1093/ofid/ofag404

**Published:** 2026-07-24

**Authors:** Brady Page, Elizabeth Moreton, David A Wohl, William A Fischer

**Affiliations:** University of California, San Diego (UCSD), La Jolla, California, USA; Health Sciences Library, University of North Carolina, Chapel Hill, North Carolina, USA; Institute for Global Health and Infectious Diseases, University of North Carolina, Chapel Hill, North Carolina, USA; Institute for Global Health and Infectious Diseases, University of North Carolina, Chapel Hill, North Carolina, USA

**Keywords:** coinfection, Crimean–Congo, Ebola, Lassa, Marburg

## Abstract

**Background:**

Concern for bacterial coinfection in patients with high consequence viral infections (HCVIs) has made empiric antibiotic administration the standard of care. However, the incidence, microbiology, and clinical significance of bacterial coinfection in HCVIs remain unclear.

**Methods:**

We conducted a systematic review of case reports, cohort studies, and cross-sectional analyses describing bacterial coinfection in Ebola virus disease (EVD), Marburg virus disease (MVD), Crimean–Congo hemorrhagic fever (CCHF), and Lassa fever (LF). Data were extracted on study design, setting, diagnostic methods, pathogens, antibiotic use, and outcomes.

**Results:**

Thirty publications met inclusion criteria. Bacteremia was most commonly reported among patients with CCHF (*n* = 46), followed by EVD (*n* = 16), MVD (*n* = 16), and LF (*n* = 2). Of 27 EVD patients treated outside Africa, 7 (26%) had bacterial coinfection. Across HCVIs, bacteremia was detected a median of 11 days after symptom onset (range 0–23) and was associated with features of sepsis. Pathogens included enteric flora, healthcare-associated organisms, opportunistic pathogens, and zoonotic bacteria—most notably *Brucella* spp. in CCHF. Ceftriaxone was the most common empiric antibiotic but is predicted to have activity against a minority of bloodstream isolates. Mortality among coinfected patients with EVD or MVD was similar to those without bacterial coinfection, whereas coinfected CCHF patients had higher mortality than those without coinfection (15.5%; 95% CI 8.0%–25.9% vs 5.7%; 95% CI 5.1%–6.4%).

**Conclusions:**

Despite being a major concern driving empiric antibiotic use, bacterial coinfection in HCVIs remains poorly described, precluding determination of its incidence or clinical significance. Prospective studies with standardized protocols for bacterial diagnosis and characterization of antimicrobial resistance are needed to guide antimicrobial strategies.

## Teaser

Bacterial coinfection in Ebola, Marburg, Crimean–Congo hemorrhagic fever, and Lassa fever is poorly characterized, likely underdiagnosed, and variably reported. Limited data suggest late-onset bacteremia, diverse pathogens, and inadequate empiric coverage, highlighting the need for standardized diagnostics and locally informed antimicrobial strategies.

Since the mid-20th century, the global community has experienced an increase in the frequency of outbreaks caused by emerging and re-emerging high-consequence viral pathogens, many with high mortality rates and limited available countermeasures [1]. Recent and ongoing epidemics—including those caused by Zaire ebolavirus (EBOV), Marburg virus (MARV), Crimean–Congo hemorrhagic fever virus (CCHFV), and Lassa virus (LASV)—underscore the expanding geographic reach and clinical severity of these high-consequence pathogens, reinforcing the urgent need to improve prevention, diagnostics, and therapeutic strategies [2–5].

Despite growing clinical experience with these pathogens, the mechanisms that drive progression to critical illness and death remain incompletely understood [6]. Clinical presentations span a wide spectrum ranging from asymptomatic or mild illness to fulminant disease marked by multiorgan dysfunction and death [7, 8]. Although several factors have been associated with mortality in these infections—including advanced age, hemorrhage, viral load, time from symptom onset to treatment, and organ dysfunction (eg, kidney failure, hepatitis, and acute respiratory distress syndrome)—there remains limited mechanistic insight into the biological processes underlying severe disease [6, 9–11].

Clinical observations suggest that some patients with severe Ebola virus disease (EVD), Marburg virus disease (MVD), Crimean–Congo hemorrhagic fever (CCHF), or Lassa fever (LF) develop a fatal sepsis-like syndrome late in their clinical course. In some cases, this deterioration occurs despite initial improvement and declining viral loads and is characterized by progressive neutrophilia and multiorgan failure, raising concerns for concomitant or secondary bacterial infection [12–14]. Several factors may predispose patients with these high consequence viral infections (HCVIs) to bacterial infection. Profound immune dysregulation during acute infection, including simultaneous immune activation and functional immunosuppression, may increase susceptibility to secondary infection from environmental or commensal organisms via translocation across gastrointestinal or respiratory mucosal barriers [15–18]. In addition, nosocomial bacterial infection during hospitalization is a substantial risk in the settings where most HCVIs are managed [19]. Lastly, the endemic ranges for many HCVIs overlap with those of several important bacterial zoonoses, which may be acquired prior to hospital admission [20–22].

Because of these concerns regarding bacterial coinfection, international guidelines commonly recommend empiric broad-spectrum antibiotics as part of supportive care for patients with suspected or confirmed HCVI [6, 13, 14, 19, 23, 24]. Although this recommendation is based on reasonable concern for secondary bacterial infection, the universal use of antibiotics in the absence of robust epidemiologic antimicrobial resistance data carries important risks, including inadequate pathogen coverage, organ–specific toxicities, selection for antimicrobial resistance, and the development of *Clostridioides difficile* colitis, which may be particularly consequential in patients with HCVIs [25–31].

Despite longstanding clinical concern that bacterial coinfection may influence the pathophysiology and outcomes of EVD, MVD, CCHF, and LF, key gaps remain in our understanding of its true incidence, its association with disease severity, and optimal diagnostic and therapeutic approaches. This systematic review addresses these gaps by synthesizing published data on (1) the incidence of bacterial coinfection in EVD, MVD, CCHF, and LF, (2) the bacterial organisms implicated, and (3) the impact of bacterial coinfection on mortality, where available. We further highlight critical gaps in the literature and propose priorities for future investigation aimed at informing antimicrobial stewardship and improving patient outcomes in the setting of increasingly frequent and severe outbreaks.

## METHODS

In accordance with PRISMA (Preferred Reporting Items for Systematic Reviews and Meta-Analyses) guidelines, we conducted a systematic review of all peer-reviewed publications reporting on EVD, MVD, CCHF, and LF with concomitant bacterial infection. The primary endpoints were the incidence of bacterial coinfection for each HCVI. Secondary endpoints included an analysis of bacterial pathogens identified, empiric antibiotic use, diagnostic technique, and where able, mortality.

### Literature Search

We performed a systematic literature search in PubMed (pubmed.ncbi.nlm.nih.gov), Embase (Elsevier), Scopus (Elsevier), Global Health (EBSCO), and Africa-Wide Information (EBSCO) databases with a data range up to 31 December 2025. The search included subject headings and keywords for Ebola, Marburg, Crimean-Congo, and Lassa viral hemorrhagic fevers plus bacterial infections. Terms for the most commonly reported bacterial coinfections were identified in the results and through the PubReMiner website and added to the search. Publications written in any language were translated and included. Animal studies were excluded. To ensure comprehensiveness, we surveyed references of included studies, citing references, gray literature, and conference abstracts. Whenever full texts of studies were unavailable, the authors of the studies were contacted. The complete search strategy can be found in the Supplementary Content.

We included all trials, case series, and case reports reporting on (1) confirmed cases of either EVD, MVD, CCHF, and LF, (2) confirmed presence of a bacterial pathogen in a normally sterile tissue or other diagnostic result that would signify infection, and (3) some identifying bacterial pathogen information. We excluded studies that only reported one of the HCVIs but without mention of bacterial coinfection. Title and abstract screening were performed by the authors. The full text of selected publications was reviewed and included if eligibility criteria were met.

### Data Extraction and Synthesis

Relevant data was extracted, including sample size, patient demographics, study location, virus species, bacterial pathogen, diagnostic technique, antibiotic use, and mortality.

### Statistical Analysis

Descriptive statistics were used to summarize study and patient characteristics. Continuous variables are reported as medians with ranges, and categorical variables are reported as counts and percentages. Exact binomial 95% confidence intervals were calculated for key reported proportions and case fatality rates (CFRs). Statistical analyses and figure generation were performed using Python version 3.11.5 (Python Software Foundation, Wilmington, DE, USA) with the pandas, NumPy, and Matplotlib libraries.

## RESULTS

### Results of Literature Search and Description of Included Studies

**Figure 1.**
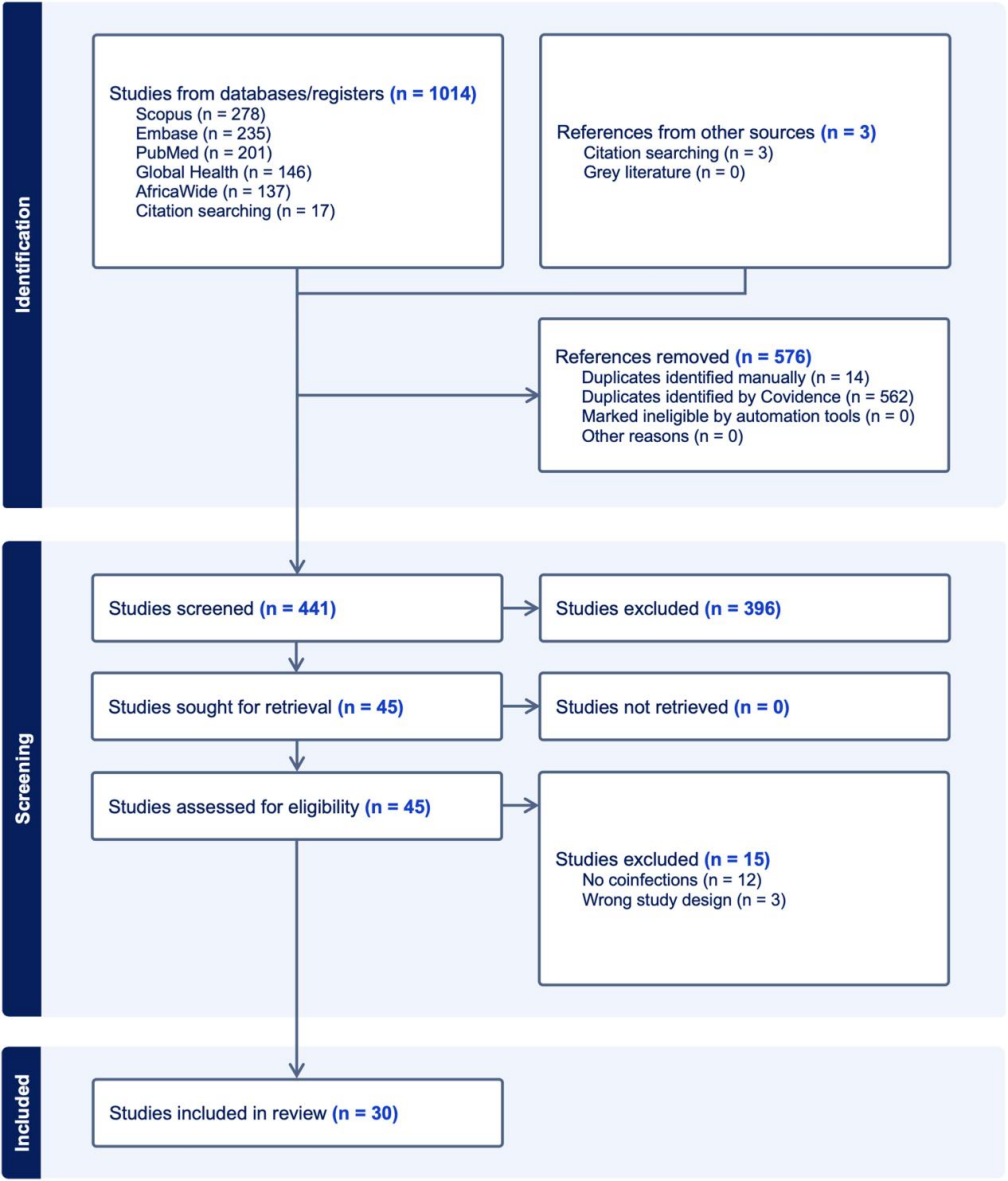
PRISMA flow diagram for study selection.

The systematic search yielded 997 references from the database searches, as well as 17 citations identified through additional searches. Duplicates were dismissed and 441 publications were included for manual screening of titles and abstracts. After identifying 396 irrelevant citations that did not include any of our primary or secondary endpoints, the full text was obtained for 45 publications, but 15 were excluded for not including a bacterial coinfection (*n* = 12) or not describing a patient case with any of our primary or secondary endpoints (*n* = 3). A flow chart of the screening process is represented in Figure 1. A total of 30 publications were advanced into full-text screening and analysis, including 13 EVD publications (9 case reports and 4 cohort studies), 1 MVD cohort study, 15 CCHF publications (11 case reports, 1 cohort study, and 3 cross-sectional studies), and 1 cross-sectional LF study. Important features from the included publications are presented in Tables 1 and 2.

**Table 1.** Cohort Studies Reporting Bacterial Coinfection in EVD, MARV, CCHF, and LF

Publication	Study Design	Population	Location	Period	Severe Viral Illness	Bacterial Coinfections	Quality Bias
**Ebola virus disease (EVD)**							
Lamb et al. J Infect. 2015;71(3):406–7 [32]	Prospective observational cohort study	Patients with confirmed EVD admitted to a military ETU	Kerry Town, Sierra Leone	2014– 2015	EVD cases: 18 EVD diagnostic: RT-PCR Age: median 31 years (range 21–63)	Individuals with coinfection: 1 Organism: coagulase-negative *Staphylococcus* Bacterial diagnostics: blood culture	◦Small single-center cohort. ◦Short blood culture incubation (2 days) may reduce sensitivity. ◦Prior antibiotic exposure in 33% of participants. ◦No outcomes data.
Carroll et al. mSphere. 2017;2(4):e00325–17 [33]	Retrospective observational cohort study	Patients with acute EVD, hospitalized survivors and fatalities, convalescent controls, and community deaths during the West African outbreak	Guinea	2013– 2016	EVD cases: 162 EVD diagnostic: RT-PCR Age: mean 28.4 years CFR: 63.4%	Organisms: *Escherichia coli*, *Pseudomonas* spp., *Klebsiella* spp., *Enterobacter* spp., *Shigella* spp., *Salmonella* spp., *Lactobacillus fermentum*, *Wohlfahrtiimonas chitiniclastica*, *Streptococcus pneumoniae*, *Myroides* spp., *Haemophilus influenzae*, *Acinetobacter baumannii*, *Providencia rettgeri*, *Ureaplasma urealyticum*, *Streptococcus sanguinis*, *Haemophilus haemolyticus*, *Campylobacter* spp., *Streptococcus parasanguinis*, *Enterococcus* spp., *Haemophilus parainfluenzae*, *Bacteroides fragilis*, and *Burkholderia multivorans* Bacterial diagnostics: deep sequencing of pooled blood	◦Retrospective analysis of diagnostic leftover samples ◦Selection bias toward patients with available RNA of sufficient quality. ◦Heterogeneous sample types (blood, plasma, swabs). ◦RNA-seq detects nucleic acid not viable organisms. ◦Limited ability to assign causality or timing of infection.
Matson et al. Open Forum Infect Dis. 2019;6(Suppl 2):S792 [34]	Retrospective observational cohort study	Patients evaluated for EVD at ELWA-3 ETU with stored triage blood samples	Monrovia, Liberia	2013– 2016	EVD cases: 235 EVD diagnostic: RT-PCR	Individuals with coinfection: 9 Organisms: *Staphylococcus aureus*, *Paenibacillus* spp., *Staphylococcus epidermidis*, and other coagulase-negative staphylococci Bacterial diagnostics: blood culture	◦Extremely small blood volume cultured (0.2 mL), reducing sensitivity. ◦Prolonged frozen storage prior to culture. ◦Single aerobic culture system. ◦Possible contamination.
Postigo-Hidalgo et al. J Infect Dis. 2025;232(1):101–112 [35]	Retrospective observational cohort study	Hospitalized patients with suspected EVD who initially tested negative for EBOV and presented with fever or hemorrhagic symptoms	Guinea	2014	EVD cases: 28 EVD diagnostic: RT-PCR	Individuals with coinfection: 1 Organism: *Haemophilus* spp. Bacterial diagnostics: high-throughput sequencing (HTS) of blood	◦16S/HTS not validated against blood culture. ◦Pooling for Illumina HTS limits patient-level attribution of infection/AMR. ◦Symptom-etiology associations are largely nonspecific.
**Marburg virus disease (MVD)**							
Nsanzimana et al. N Engl J Med. 2025;393(10):983–993 [11]	Retrospective observational cohort study	Patients with laboratory-confirmed MVD identified during the 2024 outbreak	Kigali, Rwanda	2024	MVD cases: 66 MVD diagnostic: RT-PCR Age: 61% < 35 yr, 39% ≥ 35 yr CFR: 23%	Individuals with coinfection: 16 Bacterial diagnostics: blood culture CFR: 25%	◦Timing and number of patients tested for bloodstream infection are not reported. ◦No data on the organisms causing bacteremia.
**Crimean–Congo hemorrhagic fever (CCHF)**							
Sunbul et al. J Infect. 2015;71(5):597–9 [36]	Retrospective cross-sectional study	Patients with laboratory-confirmed CCHF admitted to regional reference hospitals	Turkey	2002– 2014	CCHF cases: 4406 CCHF diagnostic: RT-PCR CFR: 7.1%	Individuals with coinfection: 42 Age: median 60 years (range 15–86) Organisms: *Staphylococcus aureus* (10), coagulase-negative *Staphylococcus* (12), Gram-negative bacilli (11), *Streptococcus* spp. (4), *Enterococcus* spp. (3), and *Acinetobacter* spp. (2) Bacterial diagnostics: blood culture on day 4 (median) LOS: median 11 days (range 2–66) CFR: 23.8%	◦Potential ascertainment bias toward severe cases managed at tertiary centers. ◦Healthcare-associated infections are likely overrepresented.
Duygu et al. J Arthropod Borne Dis. 2017 Dec 30;11(4):463–468. [37]	Retrospective cross-sectional study	Patients hospitalized with a preliminary diagnosis of CCHF and systematic evaluation for brucellosis on admission	Tokat, Turkey	2011	CCHF cases: 120 CCHF diagnostic: RT-PCR Age: mean 32.8 years (SD ±9.21) CFR: 0%	Individuals with coinfection: 5 Age: median 37.6 years Organism: *Brucella* spp. Bacterial diagnostics: Rose Bengal test, tube agglutination CFR: 0%	◦Brucellosis diagnosed primarily by serology; limited blood culture confirmation. ◦Selection bias toward patients with suspected zoonotic exposure. ◦No longitudinal follow-up. ◦Limited outcome reporting.
Öz et al. Diagn Microbiol Infect Dis. 2025;111(3):116724 [38]	Retrospective observational cohort study	Adult patients admitted with preliminary diagnosis of CCHF to a tertiary referral hospital	Sivas, Turkey	2017– 2022	CCHF cases: 885 CCHF diagnostic: RT-PCR, IgM indirect immunofluorescence CFR: 0%	Individuals with coinfection: 14 Age: median 38 years (IQR 24.5–54) Organism: *Brucella* spp. Bacterial diagnostics: Rose Bengal test, blood culture CFR: 0%	◦Retrospective single-center cohort. ◦Serologic cross-reactivity possible though cultures performed in a subset of patients. ◦No community denominator.
Topcu et al. J Clin Pract Res. 2025;47(1):68–73 [39]	Retrospective cross-sectional study	Adults (≥18 years) with samples submitted for suspected brucellosis from multiple hospital clinics	Sivas, Turkey	2017– 2021	CCHF cases: 12 CCHF diagnostic: RT-PCR	Individuals with coinfection: 12 Organism: *Brucella* spp. Bacterial diagnostics: Rose Bengal, tube agglutination, and Coombs immune capture	◦Includes only samples submitted for suspected brucellosis, so all CCHF diagnoses were already seropositive for *Brucella*. ◦Serology-based diagnosis without systematic culture confirmation. ◦CCHF testing performed only in a subset. No clinical outcome data.
**Lassa fever (LF)**							
Erameh et al. Am J Trop Med Hyg. 2025;112(6):676. [40]	Retrospective cross-sectional study	Adults and children with severe LF admitted to a single ICU	Irrua, Nigeria	2024	LF cases: 19 LF diagnostic: RT-PCR Age: mean 40.7 years CFR: 42%	Individuals with coinfection: 2 Organisms: *Escherichia coli* (1), *Klebsiella pneumoniae* (1) Bacterial diagnostics: unspecified from blood	◦Single-center ICU case series ◦Small sample size ◦Limited microbiologic detail ◦Limited demographic, clinical, and outcomes data for coinfected patients

**Table 2.** Case Reports of Bacterial Coinfection in EVD and CCHF

Publication	Location	Year	Viral Diagnostic	Patient	LOS (days)	Outcome	Viral Treatment	Bacterial Diagnosis	Organism	Antibiotic Therapy	Quality Bias
**Ebola virus disease (EVD)**											
Kreuels et al. N Engl J Med. 2014;371(25):2394–401 [41]	Sierra Leone ⇁ Hamburg, Germany	2014	RT-PCR IgM/IgG immunofluorescence	36-year old male	21	Survived	…	Prompt: leukocytosis Diagnostic: Blood culture Day of illness: 12	Gram-negative bacterium resistant to ampicillin, ciprofloxacin, and third-generation cephalosporins	Ceftazidime ⇁ Ciprofloxacin + metronidazole ⇁ Ceftriaxone ⇁ Vancomycin + meropenem	◦Organism not fully identified due to biocontainment constraints. ◦Extensive empiric antimicrobial therapy limits attribution of timing and source.
Wolf et al. Lancet. 2015 Apr 11;11;385(9976):1428–35 [42]	Sierra Leone ⇁ Frankfurt, Germany	2014	RT-PCR	36-year old male	30	Survived	FX06, Favipiravir	Prompt: leukocytosis Diagnostic: Blood culture Day of illness: 11	Coagulase-negative *Staphylococcus*	Imipenem-cilastatin ⇁ Meropenem + metronidazole ⇁ Colistin + anidulafungin + tigecycline ⇁ Linezolid + fluconazole	◦High-resource setting limits generalizability. ◦Extensive empiric antimicrobials.
Liddell et al. Ann Intern Med 2015;163:81–90. [43]	Dallas, United States	2014	RT-PCR	29-year old female	14	Survived	Brincidofovir, Convalescent plasma	Prompt: diarrhea Diagnostic: Multiplex PCR of stool Day of illness: 3	*Campylobacter* spp.	Azithromycin	◦High-resource setting limits generalizability. ◦Blood cultlures not performed due to perceived risk to laboratory workers.
Mora-Rillo et al. Lancet Respir Med 2015;3:554–62. [44]	Madrid, Spain	2014	RT-PCR	44-year old female	26	Survived	Convalescent plasma, favipiravir	Prompt: unknown Diagnostic: Multiplex PCR of whole blood days of illness: 22 and 23	Coagulase-negative *Staphylococcus*	Ceftriaxone ⇁ Piperacillin-tazobactam ⇁ Oral vancomycin + metronidazole	◦High-resource setting limits generalizability. ◦Multiplex PCR is overly sensitive compared to traditional culture, detecting contaminants
Sueblinvong et al. Crit Care Med 2015;43:2066–75. [45]	Sierra Leone ⇁ Omaha, United States	2014	RT-PCR	44-year old male	8	Died	Convalescent whole blood, ZMapp	Prompt: hemodynamic instability Diagnostic: Blood culture Day of illness: 15	*Escherichia coli*	Ceftriaxone + metronidazole ⇁ Vancomycin + meropenem	◦High-resource setting limits generalizability.
Kraft et al. Clin Infect Dis 2015; 61: 496–502 [46]	Sierra Leone ⇁ Atlanta, United States	2014	RT-PCR	43-year old male	42	Survived	TKM-100802, Convalescent plasma	Prompt: leukocytosis Diagnostic: Multiplex PCR of stool Day of illness: 7	Enteropathogenic *Escherichia coli*	Ceftriaxone ⇁ Ceftaroline + meropenem	◦High-resource setting limits generalizability. ◦The presence of pathogenic strains of *E*. *coli* in stool does not always cause disease.
O'Shea et al. Am J Trop Med Hyg. 2016;94(4):829–32 [47]	Kerry Town, Sierra Leone	2014	RT-PCR	21-year old male	26	Survived	Convalescent whole blood	Prompt: multiorgan failure Diagnostic: Blood culture Day of illness: 10	Coagulase-negative *Staphylococcus*	Ceftriaxone + metronidazole ⇁ Piperacillin-tazobactam + gentamicin ⇁ Amoxicillin-clavulanate	◦High-resource military ETU setting limits generalizability. ◦Extensive empiric antimicrobial therapy.
Wichmann et al. Med Klin Intensivmed Notfmed. 2017;112(1):38–41 [48]	West Africa ⇁ Hamburg, Germany	2014– 2015	RT-PCR	Male of unknown age	31	Survived	…	Prompt: leukocytosis Diagnostic: Blood culture Day of illness: 15	Multidrug-resistant (MDR) Gram-negative bacterium	Ceftazidime ⇁ Ciprofloxacin + metronidazole ⇁ Ceftriaxone - Meropenem	◦High-resource setting limits generalizability. ◦Extensive empiric antimicrobials. ◦Bacterial organism reported only as MDR Gram-negative.
Matson et al. Pathogens. 2020;9(6) [34]	Monrovia, Liberia	2014	RT-PCR	Female of unknown age	2	Died	…	Prompt: unknown Diagnostic: Blood culture	*Bacillus paranthracis*	…	◦Retrospective culture from stored blood. ◦No premortem antimicrobial data available.
**Crimean-Congo hemorrhagic fever (CCHF)**											
Saghafipour et al. Qom Univ Med Sci J. 2012;6(2) [49]	Qom, Iran	2011	IgM/IgG ELISA	22-year old male	…	Survived	…	Diagnostic: Tube agglutination 2-mercaptoethanol	*Brucella* spp.	…	◦Limited treatment and outcome detail
Karakeçili et al. Mikrobiyoloji bulteni. 2016;50(2):322–7 [50]	Erzincan, Turkey	…	RT-PCR IgM IFA	70-year old female	10	Survived	…	Diagnostic: Rose Bengal test Tube agglutination Coombs immune capture	*Brucella* spp.	Unknown empiric antibiotics - Rifampin + doxycycline × 8 weeks	◦Prior outpatient antibiotic use likely reduced blood culture sensitivity. ◦No ribavirin administered for CCHF.
Seifi et al. Arch Clin Infect Dis. 2016;11(3) [51]	Siahkal, Iran	…	RT-PCR	56-year old female	10	Survived	Ribavirin	Diagnostic: Serology	*Leptospira pomona*	Ceftriaxone	◦Leptospirosis diagnosis based on single low-titer serology. ◦No paired sera to confirm 4-fold rise.
Tahmaz et al. Klimik Dergisi. 2019;32(3):341–343 [52]	Antalya, Turkey	…	RT-PCR IgM ELISA	46-year old male	12	Survived	IVIG	Diagnostic: Tube agglutination Coombs immune capture	*Brucella* spp.	Unknown empiric antibiotics - Rifampin + doxycycline × 8 weeks	◦Brucellosis diagnosed serologically without culture confirmation.
Büyüktuna et al. Klimik Derg. 2020;32(3):338–340 [53]	Sivas, Turkey	2018	RT-PCR	54-year old male	16	Survived	…	Prompt: unknown Diagnostic: Tube agglutination Blood culture Day of illness: 11	*Brucella* spp.	Rifampin + doxycycline × 6 weeks	◦Empiric supportive care prior to definitive diagnosis.
Izcı et al. J Med Virol. 2021;93(6):3929–3933 [54]	Kayseri, Turkey	…	RT-PCR	18-year old female	28	Survived	Ribavirin, Therapeutic plasma exchange	Prompt: fever Diagnostic: Peripheral and catheter blood culture Day of illness: 5	*Clostridium perfringens*	Piperacillin-tazobactam + metronidazole	◦Antibiotics initiated empirically before organism identification.
Gül et al. Mikrobiyol Bul. 2022;56(2):365–370 [55]	Erzincan, Turkey	2020	RT-PCR IgM IFA	31-year old female	10	Survived	Ribavirin	Diagnostic: Rose Bengal test Coombs immune capture	*Brucella* spp.	Rifampin + doxycycline × 8 weeks	◦Serology-based diagnosis of brucellosis with negative blood culture ◦Coincident SARS-CoV-2 infection confounds clinical course
Erdoğan et al. Klimik Derg. 2023;36(1):82–85 [56]	Alanya, Turkey	2020	RT-PCR	44-year old male	15	Survived	…	Diagnostic: Tube agglutination	*Brucella* spp.	Ceftriaxone 2 g qd - Ciprofloxacin 500 mg po qd Rifampin + doxycycline × 6 weeks	◦Delayed suspicion of CCHF in a non-endemic region. ◦Empiric antibiotics before diagnosis.
Barkay et al. J Emerg Med Case Rep. 2023;14(2):33 [57]	Erzincan, Turkey	…	RT-PCR IgM IFA	35-year old female	10	Survived	…	Diagnostic: Rose Bengal test	*Brucella* spp.	Rifampin + doxycycline	◦Patient was hospitalized with a pre-diagnosis of COVID-19 and discharged once chest imaging was negative
Ajazaj-Berisha et al. Viruses. 2025;17(2) [58]	Pristina, Kosovo	2013	RT-PCR	Neonatal female	6	Died	…	Prompt: meconium aspiration Diagnostic: Blood culture Day of illness: 0	*Acinetobacter baumannii*	Unknown empiric antibiotics	◦Delayed CCHF testing after cesarean delivery. ◦Empiric antibiotics before microbiologic confirmation.
Karaşahin et al. Mikrobiyoloji bulteni. 2025;59(4):525–532 [59]	Erzurum, Turkeyu	2024	RT-PCR	42-year old female	40	Survived	…	Prompt: fever Diagnostic: Blood culture Day of illness: 11	*Staphylococcus aureus* (MSSA)	Piperacillin-tazobactam Cefazolin 1 g IV tid × 6 weeks	◦Bacterial infection occurred during recovery. ◦Abscess culture was negative due to prior antibiotics.

### Ebola Virus Disease

#### Cohort Studies

Four cohort studies evaluated bacterial coinfection in patients with EVD during the 2013–2016 outbreak in West Africa, including 1 prospective observational cohort and 3 retrospective observational cohorts conducted in Guinea (*n* = 2), Sierra Leone (*n* = 1), and Liberia (*n* = 1) using blood culture or deep sequencing methodologies (Table 1). In general, detection of bacterial coinfection in the patients described using the various methods applied was uncommon.

In the 1 prospective cohort study, Lamb et al cultured baseline blood obtained from 18 patients with RT-PCR–confirmed EVD admitted between 2014 and 2015 to a military Ebola Treatment Unit (ETU) in Sierra Leone [32]. The median age was 31 years (range 21–63), and 39% were female. Each patient had their blood cultured once on admission, except for 4 patients from whom further cultures were collected based on the development of hemodynamic deterioration or increases in C-reactive protein or peripheral white cell count. One patient (6%) had bloodstream infection detected by blood culture, yielding a coagulase-negative *Staphylococcus* isolate considered to be clinically insignificant. All patients received empiric ceftriaxone. Clinical outcomes were not reported.

In a large cohort from Guinea, Carroll et al analyzed remnant diagnostic blood samples collected on presentation from 162 patients with acute EVD between 2013 and 2016 using high-throughput RNA sequencing (RNA-seq) [33]. Although the proportion of samples with evaluable bacterial RNA was not disclosed, the authors report that *most* patients with acute EVD had extensive evidence in their blood of nucleic acid mapping to bacterial genomes, representing diverse taxa of gastrointestinal flora, environmental organisms, and respiratory pathogens (Table 1). The study reported an overall CFR of 63.4% but did not differentiate between those with and without bacterial coinfection.

A retrospective cohort from Liberia by Matson et al evaluated stored triage blood samples from 235 EVD patients admitted to the ELWA-3 ETU in Monrovia between 2013 and 2016 [34]. Blood cultures identified bacterial growth in 9 patients (4%), with reported organisms including *Staphylococcus aureus*, *Paenibacillus* spp., *Staphylococcus epidermidis*, and other coagulase-negative *Staphylococcus*. No outcomes data were reported and a very small (0.2 mL) volume of blood was used for culture.

Finally, Postigo-Hidalgo et al conducted a retrospective observational study of 550 hospitalized patients in Guinea who presented with fever or hemorrhagic symptoms and initially tested negative for EBOV by RT-PCR [35]. EBOV RNA was ultimately detected in 28 (5.1%) blood samples and high-throughput sequencing identified 16S RNA from *Haemophilus* spp. in 1 (3.6%) EBOV-positive sample, though specific patient-level attribution and organism viability could not be determined.

#### Case Reports

**Figure 2.**
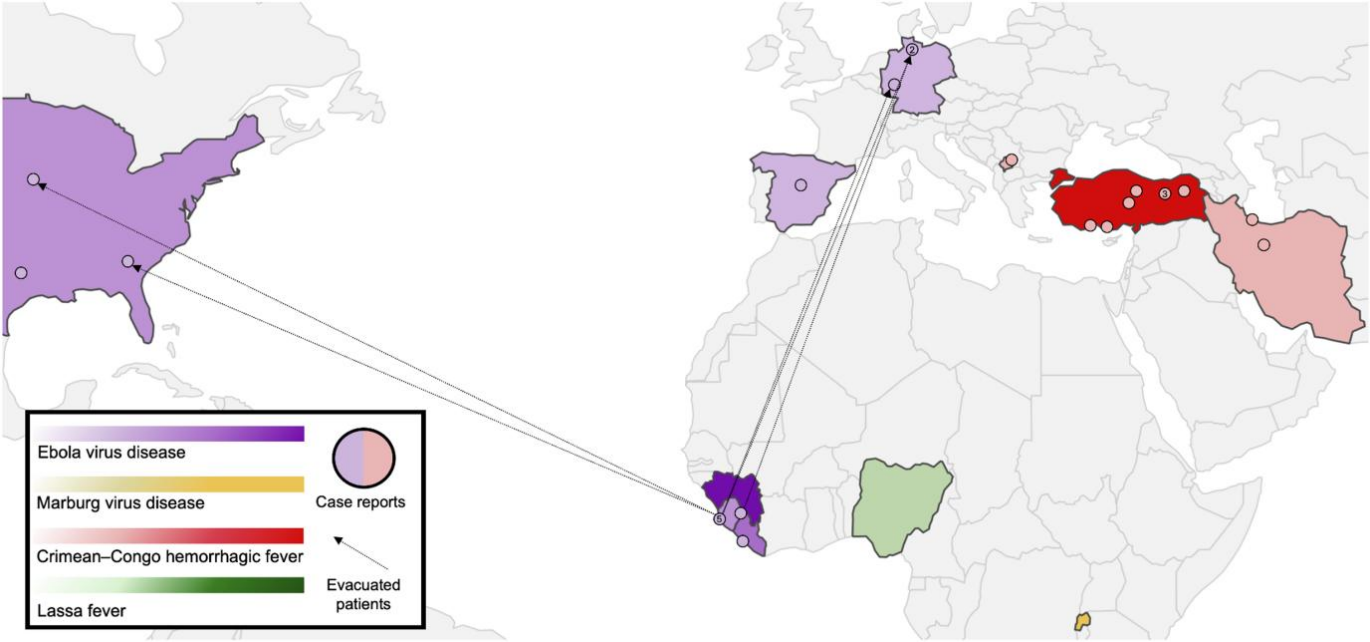
Map of Ebola virus disease, Marburg virus disease, Crimean–Congo hemorrhagic fever, and Lassa fever cases with bacterial coinfection. Countries are colored by viral infection, with darker shades representing a higher number of cases of HCVI-bacterial coinfection in published case reports, cohort studies, or cross-sectional analyses. Purple or red circles represent individual case reports of EVD and CCHF with coinfection, respectively. Three patients with EVD and coinfection were evacuated to Germany during the 2013–2016 West African Ebola epidemic: 1 from Sierra Leone and another from an unnamed country in West Africa were evacuated to Hamburg, while a third patient was evacuated from Sierra Leone to Frankfurt. Two coinfected EVD patients were evacuated from Sierra Leone to the United States during the same outbreak: 1 to Atlanta and 1 to Omaha. There was 1 autochthonous EVD case with coinfection in Dallas, United States and another in Madrid, Spain.

**Figure 3.**
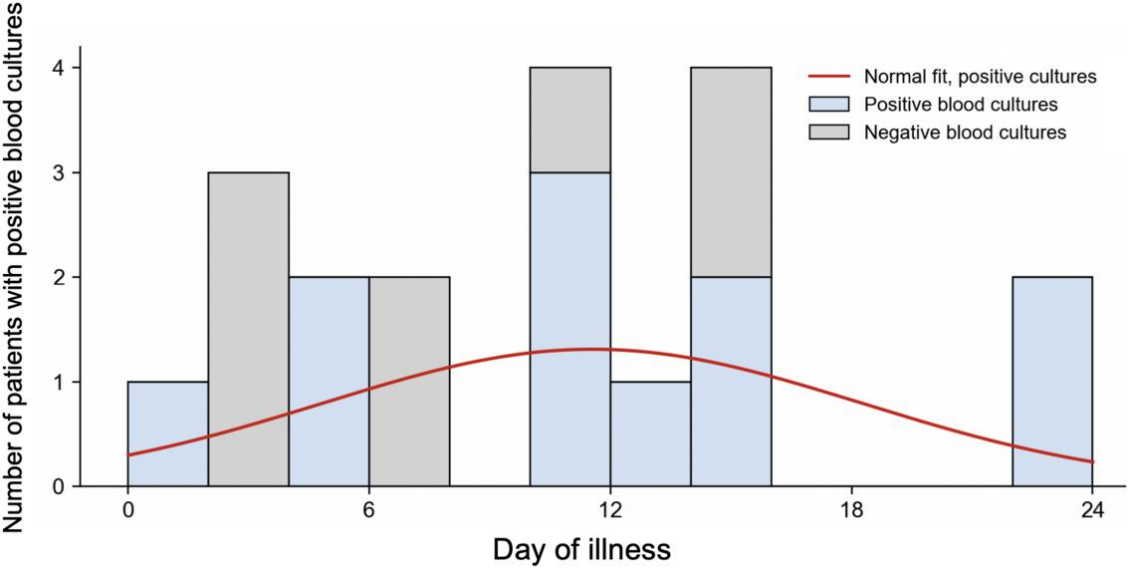
Timing of positive and negative blood culture collection for patients with Ebola virus disease and Crimean–Congo hemorrhagic fever. Blue bars represent blood cultures that were collected which ultimately resulted positive, while gray bars represent blood cultures that ultimately resulted negative. The red curve represents the fitted normal distribution of illness days on which blood cultures were collected among patients with positive blood cultures.

Between 2014 and 2025, 9 case reports describing bacterial coinfection in patients with EVD were published; all cases occurred in 2014. Of these, 3 were women (33%), the mean age among cases with available data was 36 years, and 2 patients died (CFR 22%; 95% CI 2.8%–60.0%). All but 1 patient was diagnosed with bacterial coinfection in the United States or Europe (Figure 2). Seven patients (78%) were diagnosed via blood culture and 2 via stool culture (22%). Blood cultures were positive for *Escherichia coli*, *Bacillus paranthracis*, 2 unspeciated Gram-negative bacteria, and 3 coagulase-negative *Staphylococcus* spp., which were believed to be contaminants. The 2 positive stool cultures yielded *Campylobacter* spp. and enteropathogenic *E. coli*. Blood cultures were positive on days 10–15 of illness whereas stool cultures were positive between days 3 and 7 of illness. Testing for bacterial coinfection was prompted by progressive leukocytosis (*n* = 3), gastrointestinal symptoms (*n* = 2), unknown reasons (*n* = 2), hemodynamic instability (*n* = 1), or multiorgan failure (*n* = 1). Six patients were receiving empiric antibiotics at the time of sample collection for bacterial diagnosis and all antibiotic regimens would be expected to have activity against the organisms that were ultimately identified. In addition to the negative blood cultures with available temporal data shown in Figure 3, Wolf et al report that serial cultures from blood, sputum, pleural effusion, and urine did not show growth of bacterial pathogens, Liddell et al report a negative stool culture on illness day 5, and Mora-Rillo et al report a negative stool rapid test for *C. difficile* glutamate dehydrogenase antigen and A and B toxins on illness day 21.

### Marburg Virus Disease

Only a single retrospective observational cohort study that reported bacterial coinfection in MVD was identified. Nsanzimana et al report details of the 2024 MVD outbreak in Kigali, Rwanda, including 66 patients with confirmed MVD [11]. The cohort was 32% female and 61% of patients were under 35 years old. Bloodstream infection was diagnosed in 16 patients (24%) by blood culture, and the 25% mortality rate for bacteremic patients (95% CI 7.3%–52.4%) was similar to the overall cohort mortality rate of 23% (95% CI 13.3%–34.7%). Empiric broad-spectrum antibiotics and antifungals were administered to 79% of patients, though there was no data on the specific antimicrobials administered, the number of patients cultured, the timing of culture, or the organisms isolated from blood culture.

### Crimean–Congo Hemorrhagic Fever

#### Cohort Studies

One retrospective cohort study systematically evaluated bacterial coinfection in patients with CCHF [38]. Of 885 patients with RT-PCR– or IgM indirect immunofluorescence–confirmed CCHF admitted to a tertiary referral hospital in Sivas, Turkey between 2017 and 2022, 14 (1.6%) were found to have concomitant brucellosis. Among these patients, the median age was 38 years (interquartile range [IQR] 24.5–54), 7.1% were female, and there were no reported fatalities in the entire cohort. Brucellosis diagnosis was performed using Rose Bengal serologic testing on all CCHF–positive patients and blood culture was performed on a subset of 12 patients, 3 (25%) of which were bacteremic with *Brucella* spp.

#### Cross-sectional Studies

Three retrospective cross-sectional studies evaluated bacterial coinfection in patients with CCHF, all conducted in Turkey. In the largest study, Sunbul et al performed a multicenter retrospective cross-sectional analysis of 4406 patients with RT-PCR–confirmed CCHF admitted to regional reference hospitals between 2002 and 2014 [36]. Although the number of patients who had blood cultures collected is not disclosed, culture–confirmed bacteremia was identified in 42 patients (1%), who had a median age of 60 years (range 15–86) and a higher CFR compared to the overall cohort (23.8% vs 7.1%). Bacteremia was detected a median of 4 days after admission (range 1–17) and reported organisms included *S. aureus* (*n* = 10), coagulase-negative *Staphylococcus* (*n* = 12), Gram-negative bacilli (*n* = 11), *Streptococcus* spp. (*n* = 4), *Enterococcus* spp. (*n* = 3), and *Acinetobacter* spp. (*n* = 2). The probable source of bacteremia was identified in 35 cases (83.3%), including most commonly intravenous catheters in 19/42 (45.2%), followed by the respiratory tract in 7/42 (16.7%), intraabdominal or hepatobiliary system in 5/42 (11.9%), the urinary tract in 4/42 (9.5%), and skin and soft tissue in 2/42 (4.8%) cases. Empiric antibiotics were initiated prior to blood culture collection in 50% of cases of bacterial coinfection, and 90.5% of isolates were reported to be susceptible to the empiric regimen.

Duygu et al evaluated the incidence of brucellosis among patients hospitalized in Topku, Turkey with a preliminary diagnosis of CCHF in 2011 [37]. Among 169 patients evaluated, 120 (79%) had RT-PCR–confirmed CCHF. Of these 120 patients, 5 patients (4.2%) were also diagnosed with brucellosis based on positive Rose Bengal and standard tube agglutination tests. All patients with brucellosis reported rural residence and occupational exposure to livestock or unpasteurized dairy products. All patients were treated for brucellosis and no deaths were reported.

In an additional retrospective screening study of multiple hospital clinics in Sivas, Turkey between 2017 and 2021, Topcu et al conducted a retrospective cross-sectional study of 12 279 adult patients whose blood samples were submitted for testing for suspected brucellosis [39]. Twelve patients with RT-PCR–confirmed CCHF were identified, all of whom were seropositive for *Brucella* spp. Coinfection was diagnosed using Rose Bengal testing, tube agglutination, and Coombs immune capture assays. Female patients comprised 30% of coinfections and clinical outcomes data were not reported.

#### Case Reports

Between 2012 and 2025, 11 case reports describing CCHF with reported bacterial coinfection were published (Table 2). Of the 11 cases, 7 were women (64%), the mean age was 38 years, and 1 patient died (9%; 95% CI 0.2%–41.3%). Seven patients (64%) were diagnosed by various serological techniques, 3 were diagnosed by blood culture (27%), and 1 patient was diagnosed using both techniques (9%). Serology detected *Brucella* spp. and *Leptospira pomona*. Blood cultures were positive for *Brucella* spp., *Clostridium perfringens*, *Acinetobacter baumannii*, and methicillin-susceptible *S*. *aureus*, and were positive on days 0–14 of illness. Testing for bacterial coinfection was prompted by fever (*n* = 2), meconium aspiration (*n* = 1), or unknown reasons (*n* = 1). Six patients were receiving empiric antibiotics at the time of sample collection for bacterial diagnosis and 3 antibiotic regimens would be expected to have activity against the organisms that were ultimately identified. Cases originated in Turkey (*n* = 8), Iran (*n* = 2), and Kosovo (*n* = 1). In addition to 4 patients with CCHF and *Brucella* coinfection who had negative blood cultures collected at a defined time point during their illness (Figure 3), Gül et al report that a patient with CCHF and *Brucella* coinfection had a negative blood culture collected at some point during the first 12 days of illness while Erdoğan et al report that a patient with CCHF and *Brucella* coinfection had negative urine cultures collected on illness day 2.

### Lassa Fever

A single-center retrospective cohort of 19 patients admitted with RT-PCR–confirmed LF to a reference center ICU in Irrua, Nigeria during the 2024 Lassa season was identified [40]. There were 8 fatalities (42%; 95% CI 20.3%–66.5%) in this critically ill cohort. Bacteremia was diagnosed in 2 patients (10.5%), with *E. coli* and *Klebsiella pneumoniae* identified as causative organisms. No data on demographics, treatment, or outcomes were reported specifically for the coinfected patients, though they had elevated serum levels of IL-6, procalcitonin, and C-reactive protein compared to the rest of the cohort.

## DISCUSSION

In this systematic review, we synthesized data from case reports, cohort studies, and cross-sectional analyses describing bacterial coinfection in EVD, MVD, CCHF, and LF. Bacterial coinfections were reported across all 4 HCVIs, and were most commonly documented in CCHF (*n* = 46), the 2024 MVD outbreak in Rwanda (*n* = 16), and in patients with EVD (*n* = 16), especially among those treated in the United States or Europe—settings with more advanced microbiologic infrastructure capable of performing blood cultures and other diagnostic testing. However, the majority of EVD, MVD and LF outbreaks continue to occur in resource-limited settings where systematic bacterial diagnostics are not routinely available. A recent analysis of 14 countries in sub-Saharan Africa found that only 1% of laboratories possessed the capabilities to conduct bacterial culture and antimicrobial resistance testing [60]. Therefore, regionally variable methodologies and observed differences in coinfection reporting likely reflect disparities in diagnostic capacity rather than true biologic variation between pathogens, complicating interpretation of the available data. Furthermore, the absence of standardized protocols for obtaining microbiologic cultures, including inconsistent timing, indications, and specimen collection practices, further limits comparability across studies. Nonetheless, cohort studies generally report low rates of bacterial coinfection, whereas single case reports are inherently subject to publication bias and are unlikely to reflect population-level incidence.

**Figure 4.**
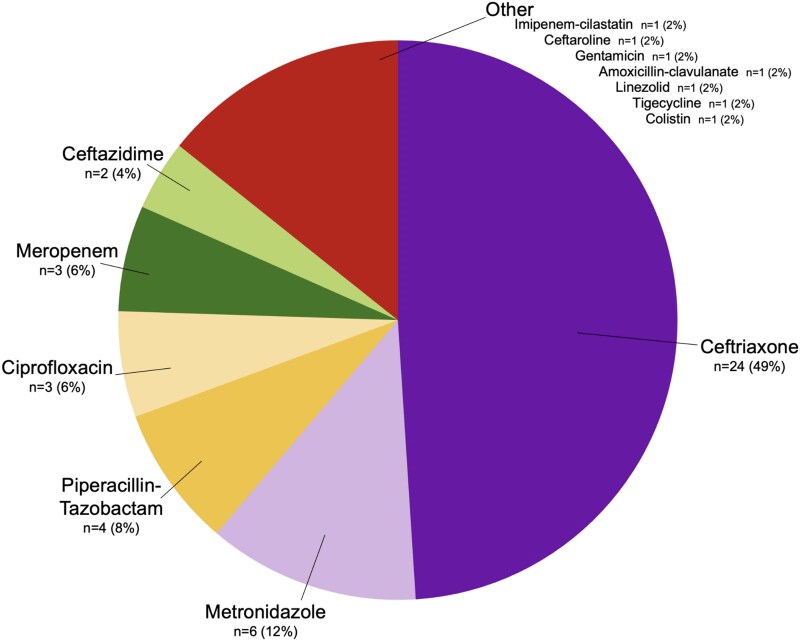
Empiric antibiotics administered to patients with Ebola virus disease and Crimean–Congo hemorrhagic fever with confirmed bacterial coinfection. Empiric antibiotics are defined as those agents administered to treat a suspected infection prior to identification of a specific pathogen, based on clinical signs, local resistance patterns, and likely organisms for that syndrome. A total of 103/146 patients (71%) with available data were identified as having received empiric antibiotics by our review. There were 49 instances of empiric antibiotic administration in which the agent used was identified: 45 in EVD and 4 in CCHF. Newer agents—such as meropenem, imipenem-cilastatin, tigecycline, linezolid, and ceftaroline—were administered once patients had been evacuated to the United States or Germany.

Empiric antibiotics were administered to 71% of patients with available data and to all but 1 EVD patient (Figure 4), creating uncertainty about whether coinfections are truly uncommon or if empiric antibiotics affected the sensitivity of bacterial diagnostics. In many instances, antibiotics were initiated prior to microbiologic sampling, reducing culture sensitivity and likely leading to underestimation of bacteremia. While this approach is consistent with international guidelines, given the difficulty distinguishing HCVI from bacterial sepsis at presentation, it complicates efforts to define the true incidence and microbiology of coinfection.

**Figure 5.**
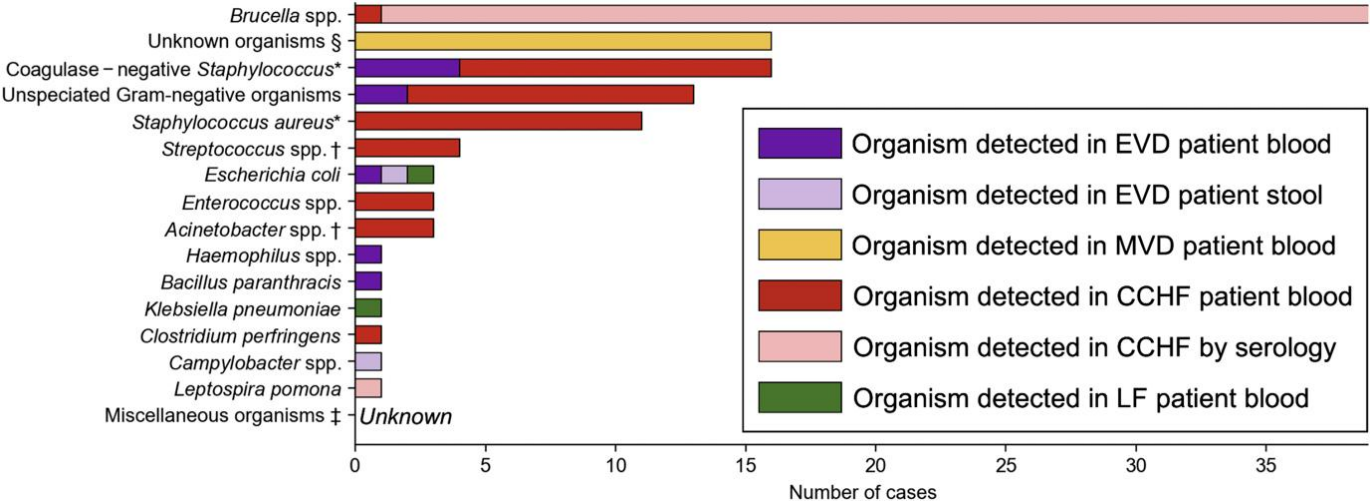
Bacterial coinfections in patients with Ebola virus disease, Marburg virus disease, Crimean–Congo hemorrhagic fever, and Lassa fever by pathogen and diagnostic modality. §There were 16 cases of unspeciated bacteremia among 66 MVD cases in the 2024 Rwandan outbreak. *Also detected in the blood of an undisclosed number of EVD patients via culture. †Also detected in the blood of an undisclosed number of EVD patients via nucleic acid sequencing. ‡Several organisms were detected in the blood of an undisclosed number of EVD patients via nucleic acid sequencing: *Escherichia coli*, *Pseudomonas* spp., *Klebsiella* spp., *Enterobacter* spp., *Shigella* spp., *Salmonella* spp., *Lactobacillus fermentum*, *Wohlfahrtiimonas chitiniclastica*, *Streptococcus pneumoniae*, *Myroides* spp., *Haemophilus influenzae*, *Providencia rettgeri*, *Paenibacillus* spp., *Ureaplasma urealyticum*, *Streptococcus sanguinis*, *Haemophilus haemolyticus*, *Campylobacter* spp., *Streptococcus parasanguinis*, *Haemophilus parainfluenzae*, *Bacteroides fragilis*, and *Burkholderia multivorans*.

Among the total of 27 patients with acute EVD who have been treated in high-resource settings, bacteremia was identified in 5 (18.5%) cases [46, 61, 62]. While some isolates (eg, coagulase-negative *Staphylococcus*) likely represent contamination, clinically significant Gram-negative bacteremia was also reported (Figure 5) [42, 44]. These findings suggest that bacterial coinfection in EVD may be under-recognized in lower-resource settings where cultures are not routinely performed.

The timing of coinfection differed between pathogens and may reflect distinct mechanisms of infection. Across HCVIs, bacteremia was detected a median of 11 days after symptom onset (range 0–23 days) (Figure 3), when clinical deterioration (eg, fever, leukocytosis, hemodynamic instability, or progressive organ failure) prompted the collection of blood for bacterial culture. In EVD, bacteremia was detected on days 10–15 of illness while in CCHF bacteremia was frequently identified earlier in the clinical course at a median of 4 days after admission (range 1–17), though the relation of this timing to the onset of illness was not disclosed [36]. While likely biased by the regional availability of diagnostics and medical evacuation of EVD patients, recognition of these temporal patterns has practical implications as clinicians should maintain high suspicion for secondary bacteremia during the second week of EVD in the setting of progressive leukocytosis, neutrophilia, or fever, whereas early bacterial diagnosis in CCHF may reflect coendemic infections present at baseline.

The bacterial pathogens identified spanned healthcare-associated, enteric, respiratory, environmental, and zoonotic taxa, encompassing both commensal flora and well-recognized human pathogens (Figure 5). The most frequently cultured clinically significant bacterium was *S*. *aureus*, consistent with hospital-acquired bloodstream infection related to vascular access devices, skin breakdown, or prolonged hospitalization [63]. *Acinetobacter* spp. are opportunistic pathogens that cause invasive infections in immunocompromised individuals, and their presence in the blood of both EVD and CCHF patients underscores the potential role of the dysregulated immune response during acute HCVI in predisposing patients to secondary infection from environmental or nosocomial organisms [15–18, 64].

Commensal gut flora—including *Enterococcus* spp., *E*. *coli*, *K*. *pneumoniae*, *B*. *paranthracis*, and *C. perfringens*—represented the second most commonly cultured pathogens from the blood of patients with HCVI. Furthermore, at least 12 additional cases of bacteremia due to unspeciated Gram-negative bacilli were reported by the included studies and nucleic acid from several members of the Enterobacterales were commonly detected among EVD patients [33]. Several HCVIs are notable for a dramatic gastrointestinal stage of illness, characterized by abdominal pain, vomiting, diarrhea, hypovolemia, and electrolyte disturbances [11, 65–67]. The detection of these organisms, together with histopathologic evidence of intestinal injury and biomarkers of gut permeability in EVD and LF, supports the biologic plausibility of mucosal barrier failure as a contributor to bloodstream infection in HCVI [68–73].

In CCHF, most reported coinfections involved brucellosis (Figure 5). Given the shared geography, seasonality, and occupational risk factors for both CCHF and brucellosis, these cases likely represent concurrent infections due to common exposures rather than a specific biologic interaction between CCHFV and *Brucella* spp [51, 74]. The frequent use of serologic testing for *Brucella* spp. in endemic regions may further amplify the apparent incidence of coinfection. Notably, available cohort data did not find an association between brucellosis and mortality in CCHF, though interpretation is limited by the unusually low reported CFRs for CCHF [37, 38].

Importantly, several identified pathogens, including *Acinetobacter* spp., *Pseudomonas* spp., multidrug-resistant Gram-negative bacilli, *S. aureus*, and *Enterococcus* spp., would not reliably be susceptible to the most commonly recommended empiric antimicrobial regimen composed of a third-generation cephalosporin plus metronidazole (Figure 4) [13, 23, 75, 76]. High regional prevalence of resistance to third-generation cephalosporins and carbapenems further limits the expected efficacy of ceftriaxone-based strategies [25, 77]. At the same time, broader spectrum agents may be unavailable or compromised by increasing carbapenem resistance [78, 79]. These findings highlight a tension between the need for empiric coverage in critically ill patients and the realities of antimicrobial resistance in many endemic regions, underscoring the need for locally informed antimicrobial stewardship strategies.

Mortality data were incompletely reported across studies, though the CFR among 9 EVD patients with bacterial coinfection was 22% (95% CI 2.8%–60.0%), lower than the overall CFR of 40% during the 2013–2016 outbreak in West Africa [80]. However, the relatively favorable outcomes observed for coinfected patients were likely influenced by the fact that 7 (78%) of these patients were treated in Europe or the United States. In contrast, the 71 coinfected CCHF patients had a CFR of 15.5% (95% CI 8.0%–25.9%), compared to 5.7% (95% CI 5.1%–6.3%) among all 5340 CCHF cases included in reviewed publications without coinfection, suggesting bacterial coinfection may be associated with worse outcomes, although the contribution of confounding factors cannot be determined from the available data. For the 2024 MVD outbreak in Rwanda, the overall CFR was 23% (95% CI 13.3%–34.7%), while patients with culture–confirmed bacteremia had a CFR of 25% (95% CI 7.3%–52.4%) [11].

This review has several important limitations, which highlight the challenge of detecting and treating bacterial coinfections in patients with HCVIs and the need for more rigorous research into this potentially life-threatening complication of these infections. The available literature is dominated by case reports and heterogeneous retrospective cohorts with inconsistent diagnostic approaches, precluding precise estimates of incidence or causal inference regarding outcomes. Microbiologic testing was rarely systematic and indications for culture were often poorly described. Additionally, empiric antibiotic use prior to sampling was likely under-reported, reduced culture sensitivity, and contributed to underestimation. Sequencing-based studies in EVD identified diverse bacterial taxa but did not consistently confirm the presence of viable organisms, limiting inference about active infection. Mortality data were incomplete and confounded by differences in supportive care, particularly among patients treated in higher-resourced settings. In addition, most EVD coinfection data was derived from the 2013–2016 West Africa outbreak, and most CCHF data originated from Turkey, potentially limiting generalizability to other regions. Finally, only a single qualifying study provided data for MVD or LF, underscoring the paucity of systematic investigation in these diseases [81, 82].

As HCVIs become increasingly common, more standardized approaches to clinical data collection and reporting are needed. Simplified case reporting developed by the World Health Organization during the COVID-19 pandemic facilitated the creation of a global database containing more than 779 million reported cases as of July 2026 [83]. A similar framework incorporating bacterial testing, antimicrobial exposures, complications, and outcomes could be adapted for HCVIs. Whereas, CCHF and LF are predictably seasonal, EVD and MVD outbreaks are episodic and often occur in settings where clinical research is challenging. Future efforts should prioritize the establishment of standardized bacterial diagnostic protocols in endemic regions where feasible, as well as the development and preapproval of protocols for prospective observational cohort studies that can be rapidly activated during HCVI outbreaks [84]. Such protocols should include standardized indications and timing for blood culture collection, documentation of antibiotic exposure prior to sampling, and linkage to detailed clinical and outcomes data. Where biosafety or infrastructure constraints preclude routine culture, molecular methods may provide complementary information but should be interpreted cautiously and validated against culture-based techniques whenever possible. Importantly, future investigations must also extend beyond West Africa and Turkey to include regions bearing the highest burden of disease—Central Africa for EVD and large areas of Europe, Asia, and Africa for CCHF—where differences in healthcare infrastructure, bacterial ecology, and antimicrobial resistance may meaningfully influence patterns of coinfection [85]. Targeted investigations into bacterial coinfection in LF and MVD are also urgently needed, as little qualifying data currently exist despite the substantial and growing burden of these diseases in endemic regions [81, 82].

## CONCLUSION

In summary, bacterial coinfection has been documented in patients with EVD, MVD, CCHF, and LF across multiple outbreak settings; however, the available evidence remains insufficient to define its true incidence or causal contribution to morbidity and mortality. These gaps reflect persistent limitations in diagnostic capacity, heterogeneous study design, inconsistent microbiologic methods, and incomplete reporting across outbreaks. The relatively high rates of coinfection identified among patients treated in high-resource settings, particularly those with EVD treated in Europe or the United States, suggest that coinfection may be a common but underdiagnosed complication of disease, with many cases emerging during the second week of illness. Furthermore, the organisms identified in this review include nosocomial and opportunistic pathogens, highlighting the importance of infection prevention and control in outbreak settings. Finally, the empiric antibiotic regimens most commonly administered during HCVI outbreaks may not reliably cover the spectrum of pathogens identified in coinfected patients, particularly in regions with high levels of antimicrobial resistance. Systematic characterization of resistance patterns, both in the community and among bacterial isolates recovered during outbreaks is essential to inform rational empiric therapy. Clarifying the true burden and microbiology of bacterial coinfection will be critical to determining whether routine broad-spectrum antibiotic administration remains justified, requires modification to account for local resistance patterns, or should be applied more selectively in patients with HCVI.

## Supplementary Material

ofag404_Supplementary_Data
