# Supplementary material for: Bacterial Infection in Ebola Virus Disease, Marburg Virus Disease, Crimean–Congo Hemorrhagic Fever, and Lassa Fever
Source: Open Forum Infect Dis. 2026 Jul 24;13(8):ofag404. doi: 10.1093/ofid/ofag404 (PMC13455356; doi:10.1093/ofid/ofag404)
Supplement: Supplementary file 1 [file ofag404_supplementary_data.docx]

**Complete search query for systematic literature review**

("Hemorrhagic Fevers, Viral"[Mesh:NoExp] OR "Filoviridae Infections"[Mesh] OR "Marburg Virus Disease"[Mesh] OR "Hemorrhagic Fever, Ebola"[MeSH] OR "Lassa Fever"[MeSH] OR "Hemorrhagic Fever, Crimean"[Mesh] OR "Marburgvirus"[MeSH] OR "Ebolavirus"[MeSH] OR "Lassa virus"[MeSH] OR "Hemorrhagic Fever Virus, Crimean-Congo"[Mesh] OR CCHF[tw] OR filovir*[ti] OR arenavir*[ti] OR arbovir*[ti] OR "marburg virus"[tw] OR "marburg fever"[tiab:~3] OR marburgvirus[tw] OR ebola[tw] OR ebolavirus[tw] OR MARV[tw] OR EBOV[tw] OR ZEBOV[tw] OR REBOV[tw] OR CIEBOV[tw] OR SEBOV[tw] OR BEBOV[tw] OR BDBV[tw] OR SUDV[tw] OR lassa[tw] OR "crimean-congo"[tw] OR "crimean fever"[tiab:~3] OR "congo fever"[tiab:~3] OR "crimea fever"[tiab:~0] OR "congo virus"[tiab:~3] OR CCHF*[tw]) AND ("Bacterial Infections"[MeSH] OR "Escherichia coli"[MeSH] OR "Staphylococcus aureus"[MeSH] OR "Methicillin-Resistant Staphylococcus aureus"[MeSH] OR "Clostridioides difficile"[Mesh] OR "Streptococcus pneumoniae"[MeSH] OR "Neisseria meningitidis"[MeSH] OR "Malaria"[MeSH] OR "Plasmodium"[Mesh] OR escherichia[tw] OR coli[tw] OR "e.coli"[tw] OR salmonella[tw] OR tuberculos*[tw] OR anthra*[tw] OR cholera*[tw] OR plague[tw] OR yersini*[tw] OR pestis[tw] OR leptospir*[tw] OR typh*[tw] OR ricketts*[tw] OR "spotted fever"[tw] OR "boutonneuse fever"[tw] OR "enteric fever*"[tw] OR "Methicillin-Resistant Staphylococcus aureus"[tw] OR MRSA[tw] OR tulare*[tw] OR botuli*[tw] OR brucello*[tw] OR "malta fever"[tw] OR "undulant fever"[tw] OR "rock fever"[tw] OR "gibraltar fever"[tw] OR "q fever*"[tw] OR "query fever*"[tw] OR legionnaire*[tw] OR "Legionella pneumophila"[tw] OR "pontiac fever"[tw] OR "coxiella burnetii"[tw] OR "bacterial meningitis"[tw] OR meningitid*[tw] OR meningococc*[tw] OR leprosy[tw] OR leprae[tw] OR "hansen disease"[tw] OR "hansens disease"[tw] OR dysenter*[tw] OR shigell*[tw] OR shiga[tw] OR bacillus[tw] OR psittac*[tw] OR "scarlet fever"[tw] OR "rheumatic fever"[tw] OR "streptococcus pyogenes"[tw] OR diphtheria*[tw] OR glanders[tw] OR melioidos*[tw] OR pseudomallei[tw] OR "whitmore disease"[tw] OR "whitmores disease"[tw] OR pertuss*[tw] OR "whooping cough"[tw] OR bartonell*[tw] OR "cat-scratch"[tw] OR "trench fever"[tw] OR "oroya fever"[tw] OR rochalimaea[tw] OR "carrion disease"[tw] OR "carrions disease"[tw] OR hemophilus[tw] OR haemophilus[tw] OR hib[tw] OR chancroid[tw] OR klebsiella[tw] OR cronobacter[tw] OR lyme[tw] OR borreli*[tw] OR tetan*[tw] OR "clostridioides difficile"[tw] OR "c. diff"[tw] OR listeri*[tw] OR syphilis[tw] OR "treponema pallidum"[tw] OR staph*[tw] OR strep*[tw] OR "bacterial pneumonia*"[tw] OR pneumoniae[tw] OR malaria*[tw] OR plasmodium[tw] OR falciparum[tw] OR vivax[tw] OR ovale[tw] OR malariae[tw] OR mycobacteri*[tw] OR "gram-negative"[tw] OR "gram-positive"[tw] OR bacteri*[ti]) AND ("Coinfection"[MeSH] OR coinfect*[tw] OR "co-infect*"[tw] OR concurrent[tw] OR coincid*[tw] OR simultaneous*[tw] OR secondary[tw] OR "bacterial infection*"[tw] OR "blood stream infection*"[tw] OR "bloodstream infection*"[tw] OR bacteremia[tw] OR bacteraemia[tw] OR septicemia[tw] OR septicaemia[tw] OR "bacterial sepsis"[tw] OR "Hemorrhagic Fevers, Viral/complications"[Mesh:NoExp] OR "Filoviridae Infections/complications"[Mesh] OR "Marburg Virus Disease/complications"[Mesh] OR "Hemorrhagic Fever, Ebola/complications"[MeSH] OR "Lassa Fever/complications"[MeSH] OR "Hemorrhagic Fever, Crimean/complications"[Mesh] OR "Malaria/complications"[MeSH]) NOT ((animal[Filter] NOT humans[Filter]) OR animal*[ti] OR zoonotic[ti] OR "nonhuman primate*"[ti] OR macaque[ti] OR monkey*[ti] OR rodent*[ti] OR "covid-19"[ti] OR "sars-cov-2"[ti])
